# How Close Do We Live to Water? A Global Analysis of Population Distance to Freshwater Bodies

**DOI:** 10.1371/journal.pone.0020578

**Published:** 2011-06-08

**Authors:** Matti Kummu, Hans de Moel, Philip J. Ward, Olli Varis

**Affiliations:** 1 Water & Development Research Group, Aalto University, Espoo, Finland; 2 Institute for Environmental Studies, VU University Amsterdam, Amsterdam, The Netherlands; University of Maribor, Slovenia

## Abstract

Traditionally, people have inhabited places with ready access to fresh water. Today, over 50% of the global population lives in urban areas, and water can be directed via tens of kilometres of pipelines. Still, however, a large part of the world's population is directly dependent on access to natural freshwater sources. So how are inhabited places related to the location of freshwater bodies today? We present a high-resolution global analysis of how close present-day populations live to surface freshwater. We aim to increase the understanding of the relationship between inhabited places, distance to surface freshwater bodies, and climatic characteristics in different climate zones and administrative regions. Our results show that over 50% of the world's population lives closer than 3 km to a surface freshwater body, and only 10% of the population lives further than 10 km away. There are, however, remarkable differences between administrative regions and climatic zones. Populations in Australia, Asia, and Europe live closest to water. Although populations in arid zones live furthest away from freshwater bodies in absolute terms, relatively speaking they live closest to water considering the limited number of freshwater bodies in those areas. Population distributions in arid zones show statistically significant relationships with a combination of climatic factors and distance to water, whilst in other zones there is no statistically significant relationship with distance to water. Global studies on development and climate adaptation can benefit from an improved understanding of these relationships between human populations and the distance to fresh water.

## Introduction

Access to freshwater is of crucial importance to humans. Traditionally, people have inhabited places close to rivers or lakes to ensure water supply for several purposes, including household water supply and water for agriculture and livestock [1]. Human population has increased rapidly during the past century, from 1.6 billion in 1900 [2] to 6.9 billion in 2010 [3]. Over the same period, the percentage of the global population living in urban areas has increased from around 16% in 1900 (i.e. 0.3 billion people) [2] to over 50% in 2010 (i.e. 3.5 billion) [4]. Over time, the relationship between human populations and freshwater bodies – and the direct dependence of humans on them – has changed, due to physical (e.g. pollution of water bodies), socioeconomic (e.g. increased population, urbanisation, and economic development), and cultural (e.g. aesthetic preferences and traditional habits) factors [5].

It could therefore be argued that today, in many parts of the world, the geographical distance to a freshwater source is not as vital for everyday survival as it was in the past. Recent technological developments have made it possible to pump groundwater from hundreds of metres below the ground and to convey it over long distances at reasonable cost through pipes and canals [6]. In addition, water can be purified efficiently and desalinisation is increasingly carried out in various arid areas [7].

However, despite these technological developments, which have ensured clean water supply for large numbers of the world's population, over 800 million people still live without improved sources (as in the WHO definition) of drinking water [8]. This development deficit is in part due to lack of investments required to implement such measures [9], either due to a lack of financial resources or other factors such as lack of institutional capacity, political will, and war. Hence, almost one billion people collect their water from distant, unprotected sources [8]; for these people the geographic distance to water bodies is still of vital importance. For many others, who are supplied with clean water, the proximity to rivers and lakes remains an important issue for aesthetic, cultural, and other reasons [1]. A short distance to water is, however, not always a positive factor. For example, in flood-prone agricultural areas (such as the Lower Mekong floodplains and large parts of Bangladesh), annual flooding may be essential for agriculture and fisheries, but living too close to the river can make populations vulnerable in the event of an extreme flood [10].

Many of the key factors that enable a good supply of water are unevenly distributed among the global population, such as: wealth [11], [12], human population [3], [13], and water resource availability [14]–[16]. Densely populated areas often do not overlap with areas that are water-rich [17]. This population pressure is projected to increase further in most countries [3] and the changing climate is also expected to increase the pressure on water resources in the future [14]–[16]. Hence, there is an increasing recognition of the need to adapt to these changes in both socioeconomic and physical drivers [18]. Global studies on climate adaptation and development would benefit from an improved understanding of the relationship between human populations and the distance to freshwater.

However, to the best of our knowledge there are no such comprehensive assessments of relationships between human populations and the distance that they live from freshwater bodies. This is despite the availability of high resolution population density datasets [13], [19] which have, in recent years, led to advances in studies examining other factors responsible for the geographical distribution of people around the globe. Examples of such factors include: urban centres [12], sea coasts [20], volcanism [21], and biodiversity [22].

In this paper we examine relationships between population density and the distance to surface freshwater bodies, in order to address the following research goals:

1. Assess the distance of land, and of human populations, to surface freshwater bodies.
2. Explore statistical relationships between population density, land distance to water, and climatic and physical factors.
3. Explore spatial relationships between population distance to water and water shortage.
4. Discuss how these insights can assist research on adaptation and development.

## Materials and Methods

In this research we examined the distance of human populations to freshwater bodies (rivers or lakes) using the population geographical Euclidean distance. This represents the closest distance of a freshwater body, in a straight line, from an inhabited area. The analyses could also have been carried out based on the closest upstream freshwater body, i.e. calculating the distance to a freshwater body from which water could be channelled by gravity. Often, however, people depending on freshwater bodies do not have the possibility to direct water through pipes or canals, but instead walk to or pump up the water according to their needs.

Naturally, some kind of weighting factor could also be introduced, as was done by the *World Bank* [12] in their study of travel times to urban centres. In the World Bank study, the travel time was calculated based on factors such as terrain, road class, and transportation options. However, we used the population geographical Euclidean distance method because the weighting factors would vary significantly depending on the use of water, and few data are available at the global scale for developing such factors.

Distance to water was first calculated on a global grid at a resolution of 1 km×1 km. For each grid-cell we calculated the average distance of each land cell to its closest freshwater body, referred to here as *land distance to water* (*dw_land_*). The results of the *dw_land_* were used to assess the population distance to the closest freshwater body, referred to here as *population distance to water* (*dw_pop_*); this was carried out at various geographical scales (e.g. administrative, physical). We also assessed the *dw_pop_* for different classes of population (urban, peri-urban, rural) and freshwater bodies (lakes and three classes of rivers). For calculating the median *dw_pop_* for different scales, we used the population as weighting factor for the *dw_land_* data: we first sorted the cells by distance and then calculated the cumulative population. The median *dw_pop_* was the distance corresponding to 50% of the cumulative population in the list.

In the rest of this section we describe the methods used in more detail. Firstly, we describe the data sources and their preparation for use in our study. Secondly, we describe the geographical scales on which we carried out the analyses. Finally, we describe the methods used to analyse the data.

### Data preparation

The data used in this study can be roughly divided into four sorts: population, freshwater bodies, climate, and geographical boundaries (see Table 1).

**Table 1:** List of the spatial data used in the analyses with source and form of data.

	Indicator/Index	Year	Source	Form of data	Notes
**POPULATION**	Population density	2007	LandScanTM 2007 [13]	Raster	Global spatial data with 30″ resolution (∼1 km at the equator).
	Urban extent	2002	MODIS 500 m urban extent map [24]	Polygon	Global spatial data with 500 m resolution.
	Peri-urban extent	2005	GRUMP dataset [25]	Polygon	Global spatial data with 30″ resolution (∼1 km at the equator).We derive peri-urban area from this dataset as described in section 2.1.1.
**WATER FEATURES**	Lakes	2001	GLWD dataset [28]	Polygon	Lake and reservoir classes of the GLWD data. Global extent with resolution of ∼1∶1,000,000.
	Large rivers	2001	GLWD dataset [28]	Polygon	River class of the GLWD data. The spatial reference of the GLWD dataset is the VMAP0 data, and thus it is compatible with the medium and small river datasets. Global extent with resolution of ∼1∶1,000,000.
	Medium rivers	1980	World Data Bank II dataset [29]	Line	The WDB II dataset was used to select the rivers from VMAP0 dataset to represent the medium rivers. Global extent with resolution of ∼1∶3,000,000.
	Small rivers	2001	VMAP0 dataset [26]	Line	River features that were not included in medium river class (see above). Global extent with resolution of ∼1∶1,000,000.
**CLIMATE**	Temperature	1960–1990	WorldClim v1.4 [38]	Raster	Global spatial data with 30″ resolution (∼1 km at the equator).
	Precipitation	1960–1990	WorldClim v1.4 [38]	Raster	Global spatial data with 30″ resolution (∼1 km at the equator).
	Available water resources per capita	2005	Kummu et al. [17]	Polygon	Available water resources per capita calculated at FPU scale.
	Aridity index	1950–2000	Trabucco and Zomer, [39]	Raster	Global spatial data with 30″ resolution (∼1 km at the equator). Based on monthly average data for period 1950–2000.
**GEOGRAPHICAL BOUNDARIES**	Country boundaries	2001	VMAP0 dataset [45]	Polygon	Country boundaries with resolution of 1∶1,000,000.
	Regional boundaries	2000	Modified from UN [33] by Kummu et al. [17]	Polygon	Globe is here divided into 12 regions.
	FPUs	2002	Modified from original FPUs [34], [35], [36] by Kummu et al. [17]	Polygon	FPUs divide the world into 281 sub-basins, each sub-basin representing a hybrid between river basins and economic regions.
	Climate regions	1975–2005	Rubel and Kottek [37]	Polygon	The average Köppen-Geiger climate classification for the year 1975–2005.

Note: GRUMP stands for Global Rural-Urban Mapping Project; GLWD stands for Global Lake and Wetland Database; MODIS for Moderate Resolution Imaging Spectroradiometer; VMAP0 for Vector Map Level Zero; and FPU for Food Production Unit.

#### Population data

Of the available population density datasets [2], [13], [19], we found the LandScanTM 2007 data [13] (see Supporting Information S1) to be the most suitable for our analysis as it provides information at the most spatially disaggregated level. LandScanTM has a resolution of 30″ (∼1 km at the equator), and the population distribution is based on census data compiled using a multi-layered spatial modelling approach [13]. The main input data are: census information; administrative boundaries; land cover; coastlines; elevation; and imagery [13]. According to the documentation of the dataset [13], the distance to water was not part of the modelling parameters. Therefore, the data are not biased in that sense and can be used for our analysis.

The LandScanTM 2007 dataset does not, however, provide any delineation between urban and rural population. We therefore used two separate datasets to identify the urban, peri-urban, and rural areas (Table 1). According to Potere et al. [23], the MODIS 500 m resolution global urban map [24] has the highest accuracy for mapping urban areas. Therefore, we selected this dataset to identify urban extent. We then used the GRUMP dataset [25] (which was assessed by Potere et al [23] as the least accurate presentation of urban settlement), together with MODIS 500 m data, to identify the peri-urban areas. The peri-urban area is here defined as the area of the GRUMP dataset that is not covered by the MODIS 500 m urban extent area. The area that is covered by neither the MODIS 500 m global urban map nor by the GRUMP data is defined as rural area (Supporting Information S1).

#### Data for freshwater bodies

For our analysis we used four classes of freshwater bodies, namely: lakes, large rivers, medium rivers, and small rivers (Table 1; Supporting Information S1). From here on we refer to these classes as water feature groups (WFGs). The spatial data for these water features are based on the VMAP0 (Vector Map Level Zero) dataset [26]. Only perennial water bodies were included in the analysis; wetlands and seasonal rivers were excluded. Of course, in some regions populations do rely on these ephemeral water sources; for example, they have determined the seasonality in farming in the Middle East for millennia [27].

The freshwater bodies were mapped using VMAP0 polygon data, which have a scale of approximately 1∶1,000,000 [26]. From the VMAP0 data, we extracted permanent lakes and large rivers using the Global Lake and Wetland Database (GLWD) [28]. In the latter database, large rivers are derived from the Level 2 data of GLWD (i.e. GLWD-2); this dataset contains the shoreline polygons of permanent open water bodies with a surface area ≥0.1 km^2^. The medium and small rivers were extracted from the VMAP0 data using the World Data Bank II (WDB II) dataset [29]. This datasets has a resolution of 1∶3,000,000; those rivers identified in the WDBII were extracted from the VMAP0 data and classed as medium rivers. The remaining rivers in VMAP0 (i.e. those that were not classed as large or medium rivers) were then classed as small rivers.

The VMAP0 river network is homogenous for most regions. However, for parts of South America and Asia, there are some differences in the level of detail in the mapping of the network. Despite this shortcoming, we believe that VMAP0 is still the most suitable dataset to be used in this kind of analysis. The recent HydroSHEDS (Hydrological data and maps based on Shuttle Elevation Derivatives at multiple Scales) data [30] have higher accuracy than the VMAP0 data, but the HydroSHEDS data do not cover the entire globe (the dataset cover only areas south from Latitude 50°N), and thus the dataset is not suitable for this study.

Due to data availability, the small streams, local surface waters, and temporal water bodies including wetlands were excluded from our analysis, although they are vital sources of water and livelihood in many parts of the world. Groundwater abstraction is also an important source of water in various regions [31], [32], but is not included in this analysis due to poor data availability. Neither does our study take into account the state of a water body in question, although water of poor quality may not be usable at all. Such information should be included in future global analyses if appropriate global data become available. The scale of the data used in the study should also be taken into account when interpreting our results.

### Geographical scales of analysis

Distance to water was first calculated on a global grid at a resolution of 1 km×1 km. The data were then aggregated to a 5 km×5 km resolution for computational reasons, before being analysed at various geographical scales, namely: Food Producing Units (FPUs); country scale; regional scale; and climate zones.

For the regional scale we used geographical boundaries that divide the globe into 12 regions (based on Kummu et al. [17], modified from UN [33]). The FPUs are based on work carried out by IFPRI (International Food Policy Research Institute) and IWMI (International Water Management Institute). These FPUs divide the world into 281 sub-basins, each sub-basin representing a hybrid between river basins and economic regions [34]–[36]. The original FPU map was slightly adjusted by Kummu *et al.* [17] to include three regions (Siberia, Iceland, and Alaska) that were collectively grouped as a ‘rest of the world’ FPU in the original data. Furthermore, some low-lying (coastal) areas and small islands, which were originally not in any FPU, were merged with the closest FPU [17]. For the climate zones, we used five different zones (equatorial, arid, temperate, cold, and polar) based on the Köppen climate zones [37].

### Data analyses

To calculate the distance to water, we first converted the maps of the four WFGs (see above and Table 1) to raster format and merged these into one layer. We then used the WFG map to calculate, for each grid-cell (including land and freshwater area), the geographical Euclidean distance to the closest water body, i.e. ‘land distance to water’ (*dw_land_*). We also calculated a ‘water feature map’ which shows, for each grid-cell, the class of the freshwater body closest to it (Supporting Information S1).

#### Population distance to water

Using the *dw_land_* dataset, combined with the population density dataset, we were able to calculate the ‘population distance to water’. This *dw_pop_* corresponds to the median distance of a person to the nearest freshwater body. We calculated *dw_pop_* for all geographical scales presented above, and for each different population and water feature group.

For each FPU we also analysed whether people lived closer to, or further from, freshwater bodies than the average *dw_land_* for that FPU. This was assessed using the ratio of *dw_pop_* over *dw_land_*, referred to hereafter as *dwr_pop/land_*.

We also analysed the average population density and cumulative population as a function of *dw_land_*. This was carried out separately for each population class and for each climate zone. With this analysis we aimed to visualise how population densities change with increasing distances to water, and to illustrate how this differs between population groups and climate zones.

#### Climatic and physical parameters affecting population distance to water

We used several climate variables (precipitation, temperature, and aridity index), together with *dw_pop_* and *dw_land_*, to explore whether population density could be explained by these physical characteristics. Bivariate correlation and multiple regression analysis tools of the SPSS programme (version 19) were used to analyse the correlations between the variables in question.

#### Distance to water and water shortage

Finally, we used estimates of water resources availability per capita from Kummu et al. [17] to examine relationships between population distance to water and water scarcity. Data for these two variables per FPU were used to construct a 3×3 matrix with the following thresholds:

Water availability per capita: chronic water shortage (<1000 m^3^/capita/yr); moderate water shortage (1000–1700 m^3^/capita/yr); and no water shortage (>1700 m^3^/capita/yr);
Population distance to water: low distance (*dw_pop_*<3.0 km); moderate distance (*dw_pop_* = 3.0–6.0 km); and high distance (*dw_pop_*>6.0 km).

## Results

**Figure 1:**
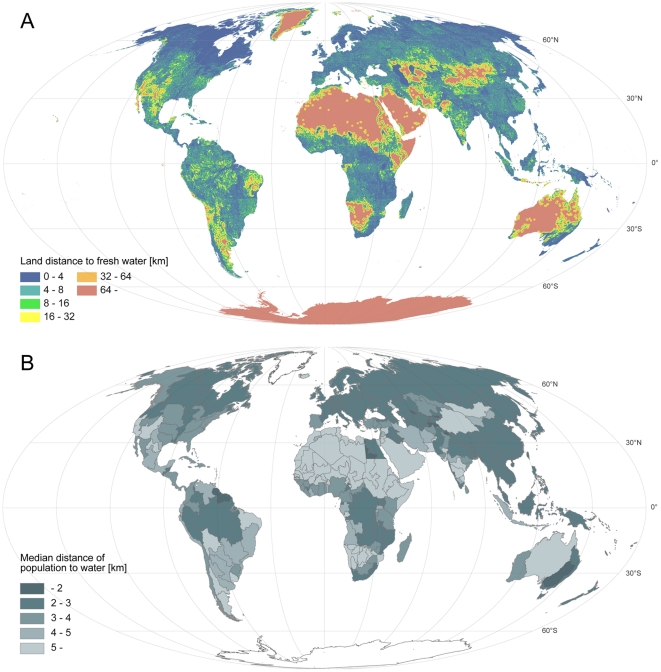
Distance to water. A: Average land distance to fresh water for each square kilometre of land (*dw_land_*). B: Median distance of population to water (*dw_pop_*) at FPU (Food Production Unit) scale.

Land distance to water (*dw_land_*) shows large spatial variations across the globe; the results are shown per square kilometre in Figure 1, panel A. Small values of *dw_land_* are found in the far northern latitudes (>50° latitude), where there are numerous lakes and rivers (Supporting Information S1), and therefore freshwater bodies are close nearly everywhere (Figure 1A). Relatively close proximity to water can also be seen in large swathes of the tropics, especially in South and Southeast Asia, parts of the Amazon basin, and tropical parts of Africa. The largest values of *dw_land_* are found in desert areas of Northern and Southern Africa, the Middle East, Central and Eastern Asia, and Australia (Figure 1A). Greenland and the Antarctic are also (at least seasonally) scarce of liquid water, although there is plenty of ice and snow.

### Population distance to water

The pattern of the median *dw_pop_* per FPU generally follows the pattern of *dw_land_*, with relatively short distances (<2 km) in northern latitudes and in the tropics, and relatively long distances (>5 km) in the more arid areas (Figure 1B). Globally, the median value of *dw_pop_* is 3.0 km (Table 2), although there are distinct differences between regions, climatic zones, and water feature and population classes. These differences will be explored in the following subsections.

**Table 2:** Population groups with the inhabited area, population, average population density, and population distance to water (*dw_pop_*).

	Inhabited area	Population	Population density^a^	Median *dw_pop_*
Population group	[10^6^ km^2^]	(×10^6^)	[persons/km^2^]	[km]
Urban	0.6 (1.1%)	1,858 (28%)	2,950	3.1
Peri-urban	2.7 (4.7%)	1,265 (19%)	480	2.9
Rural	53.4 (94.2%)	3,462 (53%)	65	3.0
**TOTAL/AVG**	**56.7 (100%)**	**6,585 (100%)**	**116**	**3.0**

Note: the total inhabited area is approximately 38% of the total surface area of the globe (see total land surface area in Table 3).

a Population density calculated by using the area of inhabited areas.

#### dw_pop_ per population type

Globally, just over half of the population (53%) lives in rural areas, whilst rural areas account for 94% of the total inhabited area (Table 2). On the other hand, whilst about 47% of the world population lives in urban and peri-urban areas (according to our division), these areas account for just 6% of the total inhabited area (being 1.6% of the total land surface area on Earth). On this global scale, the median *dw_pop_* shows very little difference between urban, peri-urban, and rural populations (Table 2); however, there are differences between regions, as will be presented and discussed later.

**Figure 2:**
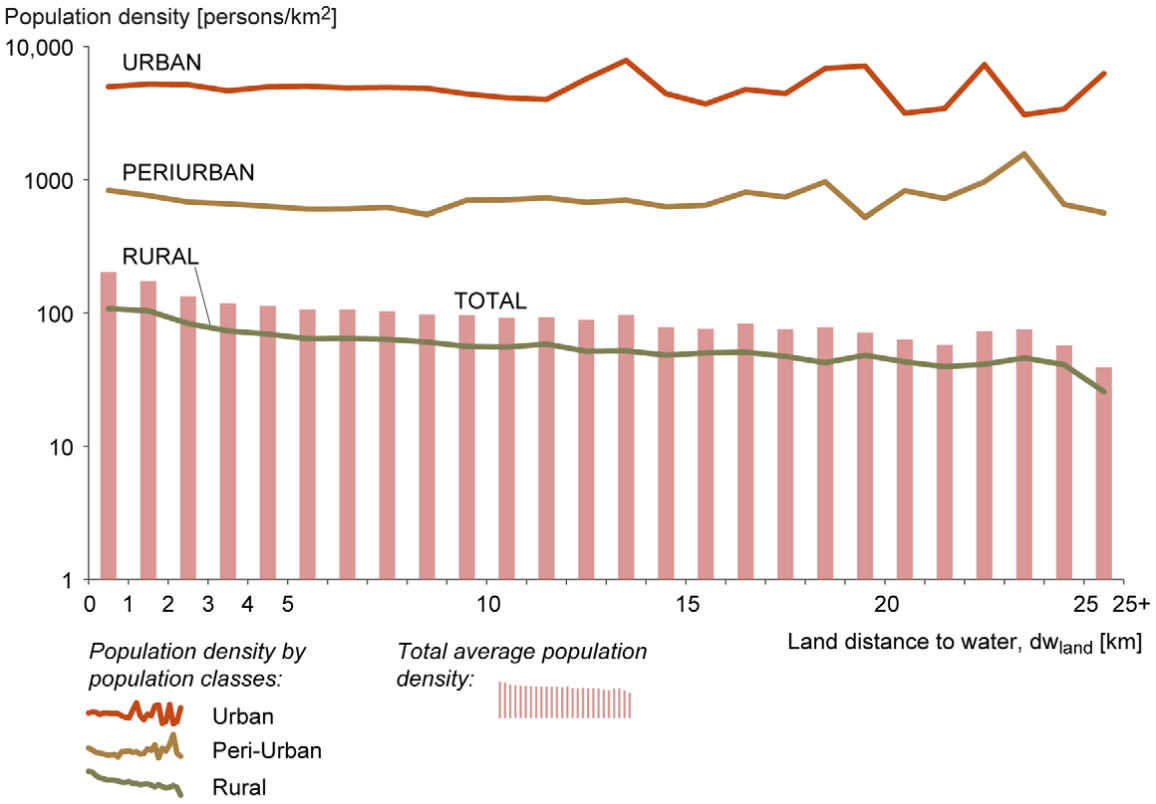
Relationship between land distance to water (*dw_land_*) and population density. The population densities for urban, peri-urban, and rural populations are presented as lines while the total average population density is presented as bars. Note: y-axis has a logarithmic scale; global average population densities are presented with a linear scale in Figure 3.

Moreover, if we examine how population density changes in relation to the *dw_land_*, we see clear differences between the population classes (Figure 2). In total, around half of the world's population lives within 3 km of a freshwater body, whilst 90% lives within 10 km. Globally, average population density gradually falls from over 150 persons/km^2^ in areas closer than 2 km to a freshwater body, to around 50–60 persons/km^2^ in areas at a distance of 25 km from a freshwater body (Figure 2, bar graph). This reduction in population density as *dw_land_* increases appears to be attributable to the situation in rural regions. Figure 2 (line graphs) shows that the population density remains rather stable as the *dw_land_* increases in urban and peri-urban areas, whilst a clear decrease is seen for rural areas. This would seem to suggest that proximity to freshwater bodies is more defining for where people live in rural areas compared to the situation in urban and peri-urban areas.

#### dw_pop_ per water feature groups

For the majority of the world population (66%) the closest water feature is a small river, while for only 6.5% of the population it is a large river (Table 3). The population density is highest, however, in inhabited areas where the closest water feature is a large river (Table 3). Based on the results derived from the datasets used, humans inhabit about 38% of the total surface area of the globe (Table 3). For those areas where a river is the closest water feature, humans inhabit over 40% of the area, while for areas where a lake is the closest water feature, only about 21% is inhabited (Table 3). This can be explained by the fact that many of the areas in which a lake is the closest freshwater feature are located in sparsely populated regions in high northern latitudes or in deserts or arctic areas (Supporting Information S1).

**Table 3:** Summary of the water feature groups (WFG) results (see also Supporting Information S2).

	Total surface area	Inhabited area	Population	Population density^a^	Median dw*_pop_*
WFG	[10^6^ km^2^]	[10^6^ km^2^]	(×10^6^)	[persons/km^2^]	[km]
Lake	32.5	6.8	829 (12.6%)	26	4.6
Large river	4.8	1.9	427 (6.5%)	90	2.2
Medium river	14.6	8.1	978 (14.9%)	67	2.9
Small river	95.8	39.8	4350 (66.1%)	45	3.0
**TOTAL/AVG**	**147.7**	**56.7**	**6,585 (100%)**	**45**	**3.0**

The *dw_pop_* stands for population distance to water.

a Population density is calculated by using the total surface area.

The median distance of population to water varies between the WFGs from 2.2 km (large rivers) to 4.6 km (lakes) (Table 3; see also Supporting Information S2). The relatively large distance to lakes can be explained by the same reasoning as the low inhabited ratio (see above). The relatively low population distance to water associated with large rivers can be related to the large population density, which appears to congregate around (inhabited sections of) large rivers.

#### dw_pop_ per climate zone

**Figure 3:**
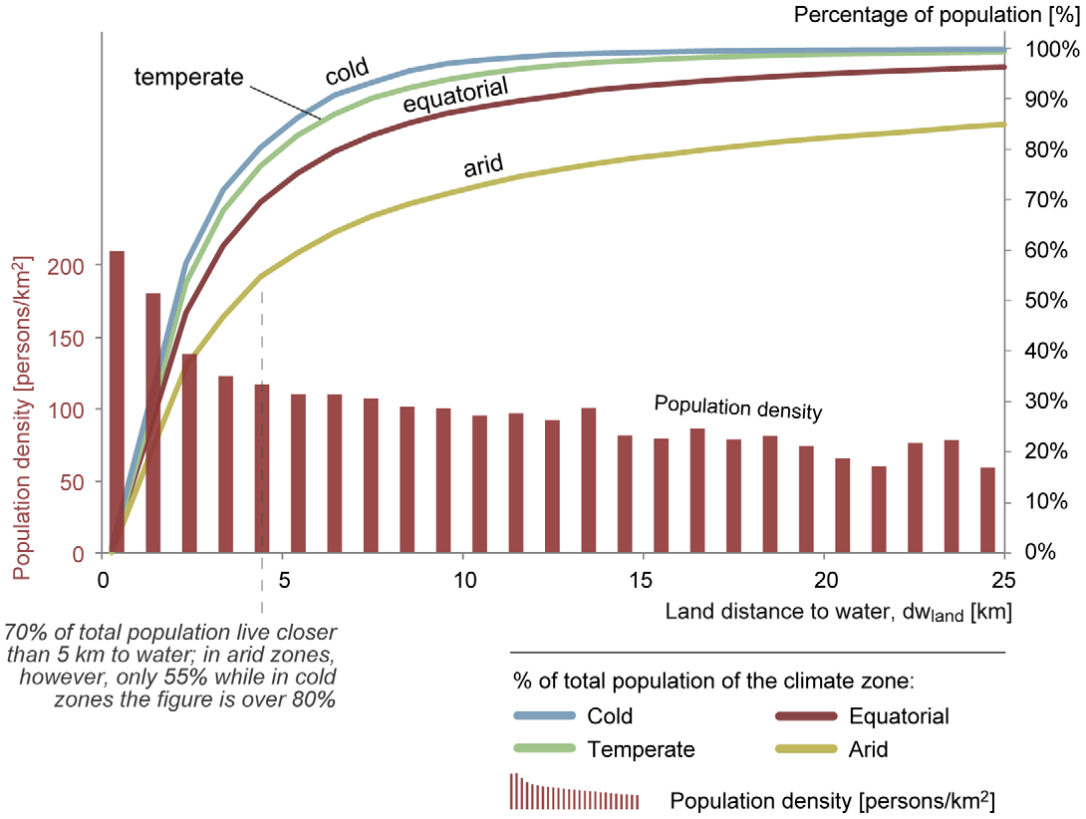
Population density vs. land distance to water (*dw_land_*) (bars) and the percentage of total population vs. distance to water (lines, differentiated between climate zones).

The decrease in global average population density as *dw_land_* increases is shown again in Figure 3. In this figure, however, the cumulative population living in different climatic zones is also shown, revealing considerable differences between the climatic regions. Whilst on a global scale about 70% of the population lives within 5 km of the closest water feature, this is around 80% for temperate and cold regions. On the other hand, only 55% of the population in arid areas lives within 5 km of the nearest water feature. Hence, in these areas, where water is already (by definition) scarce, the distance to those scarce sources is also relatively large. The median distance to water in arid zones is 4.3 km, compared to 2.8 km in cold and temperate zones.

#### dw_pop_ per administrative regions

**Figure 4:**
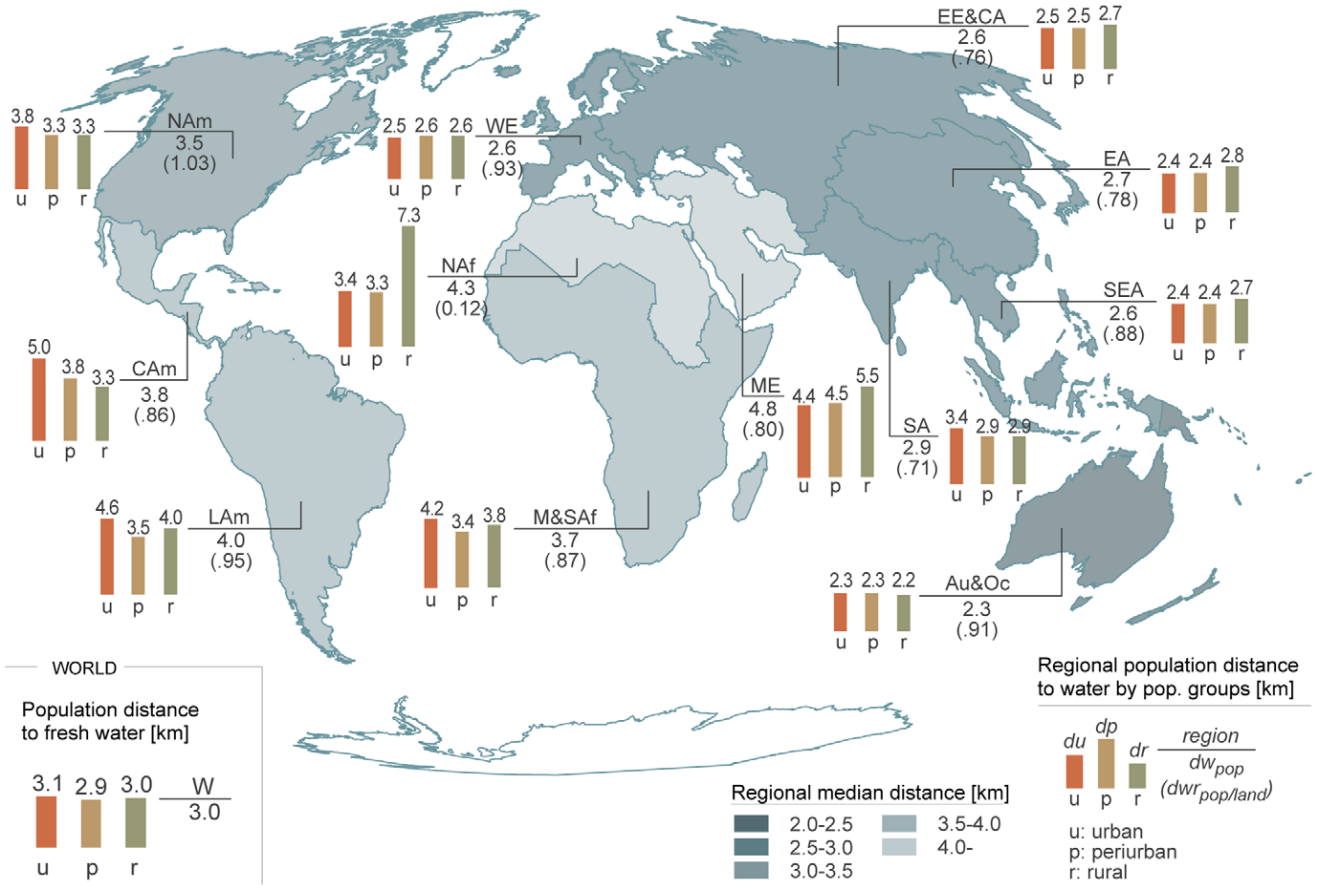
Regional results for population distance to fresh water (*dw_pop_*) with subdivision of population groups. Columns show the results per population class (urban, peri-urban, rural) while the total average distance to water is presented below the abbreviation of the region. The abbreviations for the regions are as follows: Au&Oc-Australia and Oceania; CAm-Central America; EA-Eastern Asia; EE&CA-Eastern Europe and Central Asia; SA-South Asia; LAm-Latin America; ME-Middle East; M&SAf-Middle and Southern Africa; NAf-North Africa; NAm-North America; SEA-Southeast Asia; and WE-Western Europe.

According to our analyses, people live on average closest to water in Australia and Oceania (median *dw_pop_* 2.3 km), followed by Eastern Europe, Central Asia, Southeast Asia, and Western Europe (2.6 km) (Figure 4). People in Northern Africa (4.3 km) and Middle East (4.8 km) live, on average, the furthest from water (Figure 4).

The clearest difference in median *dw_pop_* between population classes can be seen for North Africa, where the distance to water in rural areas is more than double that in urban and peri-urban areas (Figure 4). In most regions, the *dw_pop_* for urban populations tends to be rather similar to the *dw_pop_* for rural populations (difference less than 0.5 km). Interesting differences are in the Middle East and North Africa, where urban populations live closer to water than rural populations, contrasting with the Americas, where rural populations live closer to water than urban populations.

We also calculated the median *dw_pop_* for each country with more than 100,000 inhabitants. According to the results, people in Suriname live closest to water (median *dw_pop_* was 1.6 km); the median distance is also less than 2.0 km in Kyrgyzstan and Tajikistan. The people of Libya live, on average, the furthest from water (233 km). All the country results are presented in Supporting Information S2.

### Influence of climatic and physical parameters on population distance to water

#### Ratio of dw_pop_ over dw_land_

**Figure 5:**
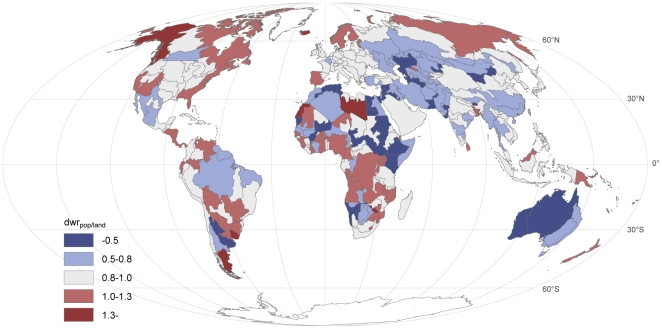
Ratio (*dwr_pop/land_*) of ‘population distance to water’ (*dw_pop_*) over the ‘land distance to water’ (*dw_land_*) by FPUs (for regional results see **Table 4**; Supporting Information S2). In areas where the ratio is smaller than 1, people live relatively close to water as the average *dw_pop_* is lower than the average *dw_land_* in that FPU. For areas with a ratio greater than 1, on the other hand, the opposite is the case and people live relatively far from freshwater sources. The thresholds are derived from the statistical analysis as follows: *dwr_pop_*_/*land*_ is between 0.5–1.3 for 95% of the cases, and between 0.8 and 1.0 in 50% of the cases (i.e. the grey values represent FPUs within this 50% interval).

As described in the methods section, the ratio *dwr_pop/land_* per FPU was used to examine whether people live closer to, or further from, freshwater bodies than the average land distance to water for that FPU. These ratios are shown in Figure 5. In large parts of the world, the population distance to water is, on average, similar to the *dw_land_*, i.e. the *dwr_pop/land_* is close to 1 (roughly one quarter of the data fall below a threshold of 0.8 while the median is 0.88; see Supporting Information S2). For many arid areas, however, the ratio *dwr_pop/land_* is relatively low (Supporting Information S2); these areas include Australia, the Sahara, and Central Asia. On average, the populations in these areas live much closer to water than the average *dw_land_* (Figure 5).

We also calculated the regional ratios of *dwr_pop/land_* per climate zone (Table 4; Supporting Information S2). Again, we see that the ratio is lowest in arid zones, except for in Southeastern Asia, where populations in temperate zones live closest to water (Table 4). For half of the regions, the ratio is highest in the temperate zone, while for others it is highest in either the cold or tropical zones (Table 4). In many regions the differences are, however, rather small. At the regional scale, ratio of *dwr_pop/land_* was smallest in Asia and largest in North America (Table 4; Supporting Information S2).

**Table 4:** Regional results for total population, population distance to water (*dw_pop_*), land distance to water (*dw_land_*), and ratio (*dwr_pop_*_/*land*_) of *dw_pop_* over *dw_land_*, for each region as a whole and by regional climate zones.

					*dwr_pop_*_/*land*_ by climate zones			
REGION	Pop.[10^6^]	*dw_pop_*[km]	*dw_land_*[km]	*dwr_pop_*_/*land*_[-]	Tropic[-]	Arid[-]	Temperate[-]	Cold[-]
Australia and Oceania	29	2.3	2.5	.91	**1.04**	**.** ***54***	.91	**.** ***65***
Central America	182	3.8	4.4	.86	**1.01**	**.** ***53***	1.02	n/a
Eastern Asia	1556	2.7	3.5	**.** ***78***	.95	**.** ***45***	.80	.89
E. Europe and C. Asia	393	2.6	3.4	**.** ***76***	n/a	**.** ***31***	.88	**.** ***78***
South Asia	1500	2.9	4.1	**.** ***71***	**.** ***71***	**.** ***51***	.88	.81
Latin America	372	4.0	4.2	.95	**1.04**	**.** ***41***	.91	**1.22**
Middle East	274	4.8	6.0	.80		**.** ***57***	.94	.82
Middle and Southern Africa	729	3.7	4.3	.87	**1.02**	**.** ***35***	1.10	n/a
North Africa	194	4.3	35.8	**.** ***12***	.84	**.** ***08***	.82	n/a
North America	333	3.5	3.4	**1.03**	.86	.85	1.01	.95
Southeastern Asia	558	2.6	3.0	.88	.89	.88	**.** ***73***	**1.02**
Western Europe	420	2.6	2.8	.93	n/a	.87	.91	**1.02**

The ratios below the 25^th^ percentile (i.e. *dwr_pop_*_/*land*_<0.8) are typed with bold italic font while the ratios above the 75^th^ percentile (i.e. *dwr_pop_*_/*land*_<0.8) are bold.

#### Statistical relationships

In order to examine statistical relationships at the FPU scale between population density and physical characteristics, we performed bivariate and multiple regressions using the SPSS software for the variables shown in Table 5. Data on precipitation and temperature were taken from the WorldClim v1.4 database [38], and refer to mean annual values for the period 1960–1990. We also used the aridity index of CGIAR [39]; this index represents the ratio of mean annual precipitation over mean annual potential evapotranspiration. The regression results are shown for the globe and per climate zone in Table 5. Bivariate regression results between all parameters (on a global scale) and multiple regression analysis for different arid regions are presented in Supporting Information S2.

**Table 5:** Results of the bivariate and multiple regression analysis.

		BY CLIMATE ZONES			
Variable	Globe(n = 285)	Tropic(n = 87)	Arid(n = 95)	Temperate(n = 55)	Cold(n = 48)
dw_land_	.152	.869	.096	.439	.818
Aridity	.017*	.169	.205	.317	.314
Prec	.002**	.152	.112	.916	.000***
Temp	.099	.100	.901	.411	.000***
dw_land_& aridity	.042*	.302	.086	.462	.512
dw_land_& prec	.010**	.266	.008**	.742	.000***
dw_land_& temp	.047*	.255	.198	.587	.001**
Aridity & prec	.010*	.358	.216	.537	.000***
Aridity & temp	.010*	.130	.448	.555	.001**
Prec & temp	.009**	.139	.239	.672	.000***
dw_land_& aridity & prec	.023	.450	.018*	.578	.000***
dw_land_& aridity & temp	.014*	.245	.146	.656	.002**
dw_land_& prec & temp	.017*	.258	.002**	.778	.000***
Aridity & prec & temp	.019	.255	.348	.741	.000***

The dependent variable was population density; the predictor(s) of each case are listed in the first column. The analysis were carried out at the FPU scale, for the whole globe, and then separately for each climate zone (grouped by spatially dominant climate zone in a FPU). Note: *dw_land_* stands for land distance to water, prec for precipitation, and temp for temperature.

*: p<0.05;

**: p<0.01;

***: p<0.001.

On a global scale, we found significant bivariate correlations between population density and both aridity and precipitation (Table 5), indicating higher population densities with higher precipitation and lower aridity. However, when performing the bivariate regressions for each climate zone individually, the only significant correlations are in the cold region, for the parameters precipitation and temperature (Table 5). Similar results were found when performing multivariate regressions using two parameters. At the global scale, all combinations of parameters are significant, but within climatic regions significant regressions were mainly found in the cold region. The only exception is the combination of *dw_land_* and precipitation, which resulted in a significant regression in arid zones.

Performing regression analyses using three parameters resulted in more interesting results. In the arid zone, adding *dw_land_* to both precipitation & temperature and to aridity & temperature resulted in significant correlation, whereas there was no significant correlation between population density and the latter pairs of variables without *dw_land_*. This indicates that population densities in arid zones are influenced by a combination of distance to freshwater bodies and precipitation/aridity. We also divided the arid zone into five geographical regions (see Supporting Information S2) and performed the same regression analyses as presented above, in order to find possible regional differences within the arid zone. We found that the correlations between *dw_land_* and population density are strongest in Northern Africa and Middle and Southern Africa (see all the results in Supporting Information S2).

Overall, it seems that in the tropical and temperate zones the concentration of populations cannot be explained by either climatic factors or the distance to freshwater bodies. In the cold zone, climate variables play a very important role, whilst in arid regions population densities can be explained by a combination of climatic factors and distance to freshwater bodies.

#### Water shortage in relation to dw_pop_

**Figure 6:**
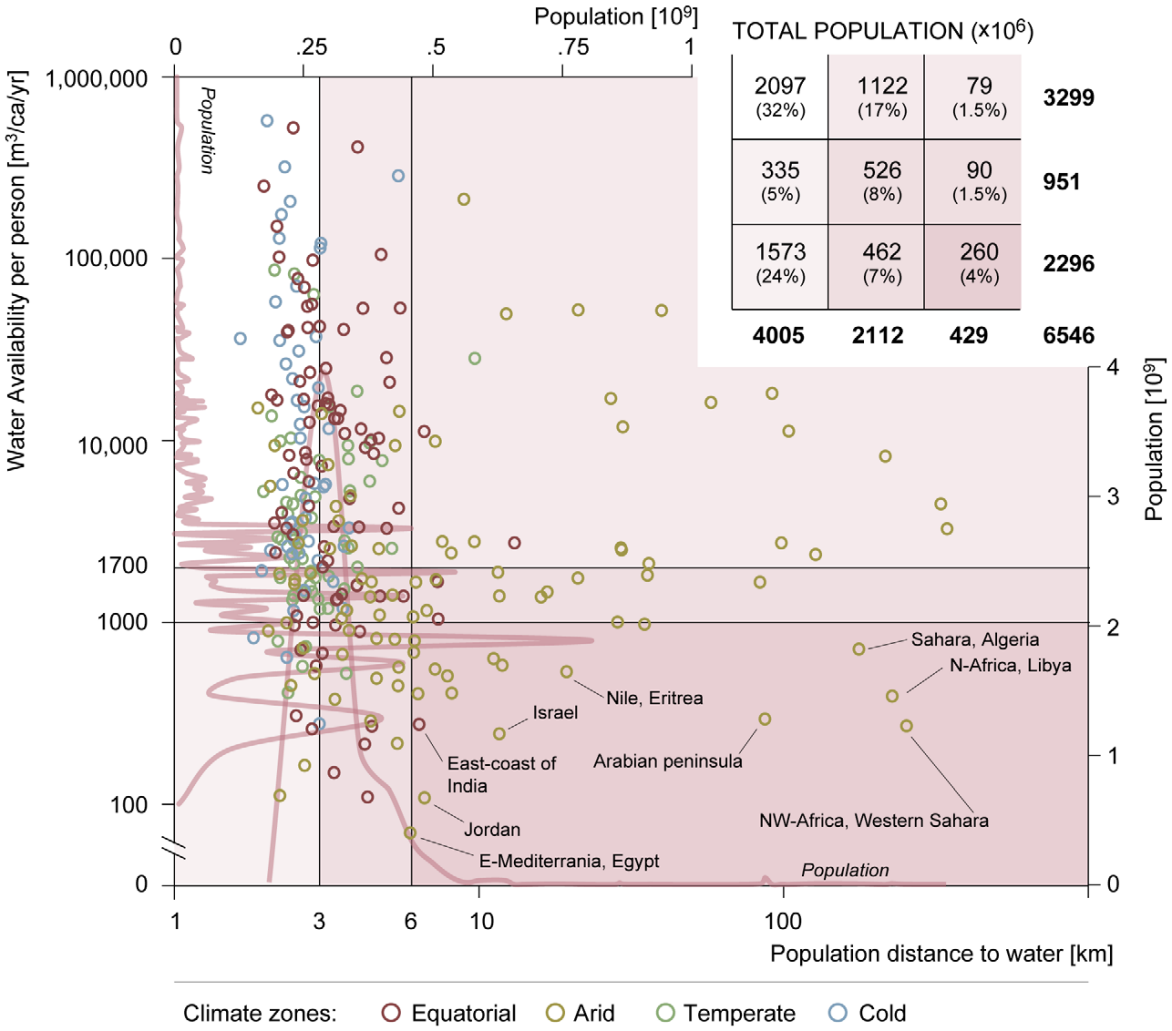
Scatter plot of population distance to water and water availability per person. Analysis scale is the FPU level. Total population in the matrix's nine areas is presented in the top right corner of the plot, the percentage of the world's total population is in brackets. The lines represent population distributions for the distance to water (lower x-axis, right y-axis) and for water availability (left y-axis and upper x-axis). These lines represent where large concentrations of people are present. Note: the left and lower axes have a logarithmic scale.

We compared our results of population distance to water per FPU with estimates of water availability per person (in the year 2005) from Kummu et al. [17]. Figure 6 shows for each FPU the water availability versus the median population distance to water. The figure is divided into nine parts of a matrix. FPUs in the lower right corner are those which suffer from both chronic water shortage and for which the average distance to freshwater bodies is large. Almost all of the FPUs found in this part of the matrix are located in arid climate zones. However, not every arid FPU with a long distance to water suffers from water shortage, as can be seen from the points in the upper right corner. In contrast, there are also FPUs that suffer from chronic water shortage whilst having a relatively low population distance to water. These are in the lower left corner and are mainly areas with high population density in parts of Europe, East Asia, and South Asia (see Supporting Information S2).

A long distance to freshwater might be an extra stress factor on top of physical water shortage for populations living in such areas. Around 70% of the population under chronic water shortage (<1000 m^3^/capita/yr) lives in areas relatively close to water (<3.0 km), while 260 million people live in areas relatively far from water (>6 km), mostly in the arid zones of Middle East and Northern Africa (Figure 6). Approximately 20% of the global population lives in areas under some kind of water shortage (<1700 m^3^/capita/yr) and further than 3.0 km (global median) from the nearest freshwater body.

## Discussion

### Major factors influencing distance to water

We found clear regional differences in the distance to which human populations live from water, with people living closest to water in high northern latitudes and parts of the tropics, due to the abundance of many rivers and lakes. Interestingly, whilst the population distance to water is generally highest in arid regions, the relative distance to water (i.e. *dwr_pop_*_/*land*_) is lowest in these regions.

There are also large differences between the different types of population groups (urban, peri-urban, and rural). Our results clearly show that, on a global scale, population density is not greatly affected by *dw_land_* in urban and peri-urban areas, whilst in rural areas there is a clear decrease in population density as the *dw_land_* to freshwater increases (see Figure 2). These global findings mask important differences between regions. We have shown that in most regions, the *dw_pop_* for urban populations tends to be rather similar to the *dw_pop_* for rural populations. However, interesting differences are found in the Middle East and North Africa, where urban populations live significantly closer to water than rural populations, and in the Americas, where urban populations live further from water than rural populations. This could be related to the fact that large cities of the Americas developed much later than many of the major cities in the old world, by which time means of transporting water from source to consumption point were more advanced.

The most distinct difference in median *dw_pop_* between population classes can be seen for North Africa, where the *dw_pop_* in rural areas is more than the double that in urban and peri-urban areas (Figure 4). This may be because in this (mainly) arid region, water bodies are more limited, thus increasing their attractiveness for human settlement, and resulting in urban areas close to them. In addition, the region contains many ancient cities where proximity to fresh water was essential for the founding of large settlements. Also, in the present day the GDP of many countries in this region is relatively low [12], meaning that high costs of water transport may make it financially prohibitive to locate cities far from freshwater bodies. On the other hand, rural populations in this region appear to live relatively far from freshwater bodies; this could have several causes. For example, in response to the arid conditions of the region, agricultural practices may have evolved to be able to make use of rainwater harvesting techniques and ground- or soil-water sources. Moreover, there are large numbers of ephemeral streams and wetlands in the region, which may be essential for rural communities. However, ephemeral water bodies and ground- or soil-water sources are not included in our analysis.

### Implications for adaptation and management

Global studies on climate adaptation and development can benefit from an improved understanding of the relationship between human populations and the distance to freshwater. For example, global estimates of the costs of adapting to climate change in the water supply sector [9], [40] have so far used decision rules on preferred adaptation options based on water availability and cost. However, such rules could be improved by incorporating spatial patterns of the distance of human populations from water. For example, in regions where people live far from surface water bodies, adaptation based on water transport may become prohibitively expensive, and groundwater use or rainwater harvesting may be more effective and/or efficient.

In this study, we have shown that populations in arid zones tend to live the furthest from freshwater bodies in absolute terms. On average, people in Northern Africa and the Middle East live furthest from water, and this is especially the case for rural populations in North Africa. Hence, when estimating global adaptation requirements and costs one must consider that long-distance transport of water from reservoirs may not be feasible in the latter. Also, between similar regions, the ability to adapt is related to financial means; in more affluent arid regions those means may be more readily available for implementing such systems, whilst in less-affluent regions a focus on smaller scale activities such as rainwater harvesting may be preferential.

Our results also show large regional differences in distance to water between urban and rural populations. Again, this is important to consider in planning integrated water management and adaptation measures as water requirements differ between urban and rural areas; globally aggregated estimates may mask these important differences.

Several studies have also shown that in many parts of the world, river runoffs, and thus water availability, are significantly related to different forms of interannual climate variability [41]–[43]. This should also be considered when designing measures for water supply; especially those people directly dependent on a distant freshwater body can be severely impacted if water availability is decreased in a given year (or several years) due to such variability.

With our analysis, we hoped to provide additional information related to ‘access to safe drinking water’, which is one of the assessment measures used by WHO (World Health Organisation). The definition of WHO changed, however, after year 2000 from ‘access to clean water’ to ‘access to improved drinking-water source’ [44]. Thus, rivers and streams are excluded from the new definition. We do believe, however, that rivers and streams are important in many ways for those 13% of the global population without access to improved drinking-water sources [8], and also to people who obtain their drinking water from secured sources but do use unimproved water sources for activities such as the washing of laundry. Thus, our results and methodology could be useful for further analysing the situation of populations in countries with poor access to water. Our results also identify regions where extra attention may already be needed to supply water given the physical shortage and relatively long distance to surface freshwater sources.

### Future research needs

The limitations of this study, discussed in the materials and methods section, give a pathway for future research needs in distance to water calculations.

The inclusion of small streams, local surface waters, springs, ground water sources, and ephemeral water bodies (including wetlands) in the calculations could better reflect the relationships between populations and fresh water, particularly in rural areas. In the present study, those water sources were excluded from the analysis due to poor data availability, but they should be included in future global analyses as soon as appropriate global datasets become available.

Water quality is also an important factor in the relationship between population and water. Poor water quality may decrease the usefulness of water, even if water would be at a close proximity, for example in many densely populated or industrialised areas. A global dataset of water quality could allow us to exclude polluted freshwater bodies from the analysis.

In this study we were not aiming to separate cultural or economic factors from physical factors when analysing distance to water. Naturally, in some parts of the world the distance to water is much more crucial for survival in everyday life, while elsewhere it may have a more aesthetic, cultural, or recreational value. More detailed analysis of these different ‘values’ of water would be an interesting addition to the work presented here. Furthermore, rapid population (and economic) growth and urbanisation have probably changed the relationship between water and human populations. Thus, an historical analysis of how the distance to water has evolved could reveal interesting regional trends.

The limitations of the study, discussed in this section and in Section 2 (Materials and Methods), should be taken into account when interpreting the results. We highly recommend limiting the use of the results to the macro-scale (i.e. regional to global).

### Concluding remarks

In this study we assessed the distance between human populations and surface freshwater bodies on a global scale. We aimed to increase the understanding of how inhabited places relate to surface freshwater bodies in different climate zones and administrative regions. Even though the population distance to water shows large variations for a variety of reasons, some general conclusions can be drawn from our results:

1. Global median population distance to water is 3 km, being almost the same in urban, peri-urban, and rural areas. The absolute distance to water is greatest in the Middle East and North Africa, and in several other areas with an arid climate. The relative distance (i.e. how close people live to water in relation to the existing water features in that region, measured here with *dwr_pop_*_/*land*_) is, however, shortest in arid zones, and particularly in North Africa.
2. The relative distance to water (*dwr_pop_*_/*land*_) correlates strongly with the aridity index, and adding distance to water in multivariate regression analyses improves the predictive power of the regression in arid zones considerably. This indicates that the distance to rivers and lakes is an important factor in determining where people live in arid zones. This effect is not present in tropical and temperate zones. We also found that the land distance to water has a stronger impact on population densities in rural areas, compared to in peri-urban and urban areas.
3. Many areas in which people live relatively far from freshwater bodies, also suffer from water shortage, i.e. the water is scarce in many ways.

Since population distance to water is a very basic element of human societies, it is of interest to both the general public as well as the scientific community dealing with natural resources management and climate change. Global studies on development and climate adaptation can particularly benefit from an improved understanding of the relationships between human populations and the distance to fresh water. For example, in regions where the population lives far from water bodies, adaptation based on water transport may become prohibitively expensive and unsustainable, and groundwater use and rainwater harvesting may be more effective and/or efficient. Our results also identify regions where extra attention may be needed to water supply in the near-term, i.e. those regions where populations live relatively far from freshwater bodies and also already suffer from water shortage.

## Supporting Information

Supporting Information S1 Supplement for Materials and methods section.
(PDF)

Supporting Information S2 Supplement for Results section.
(PDF)
