# Supplementary material for: How Close Do We Live to Water? A Global Analysis of Population Distance to Freshwater Bodies
Source: PLoS One. 2011 Jun 8;6(6):e20578. doi: 10.1371/journal.pone.0020578 (PMC3110782; doi:10.1371/journal.pone.0020578)
Supplement: Supporting Information S1 — Supplement for Materials and methods section. (PDF) [file pone.0020578.s001.pdf]

Supporting information S1: Supplement for Materials and methods section

of the article: *Kummu et al. 2011. How close do we live to water? A global analysis of population distance to freshwater bodies,* published at PLoS ONE

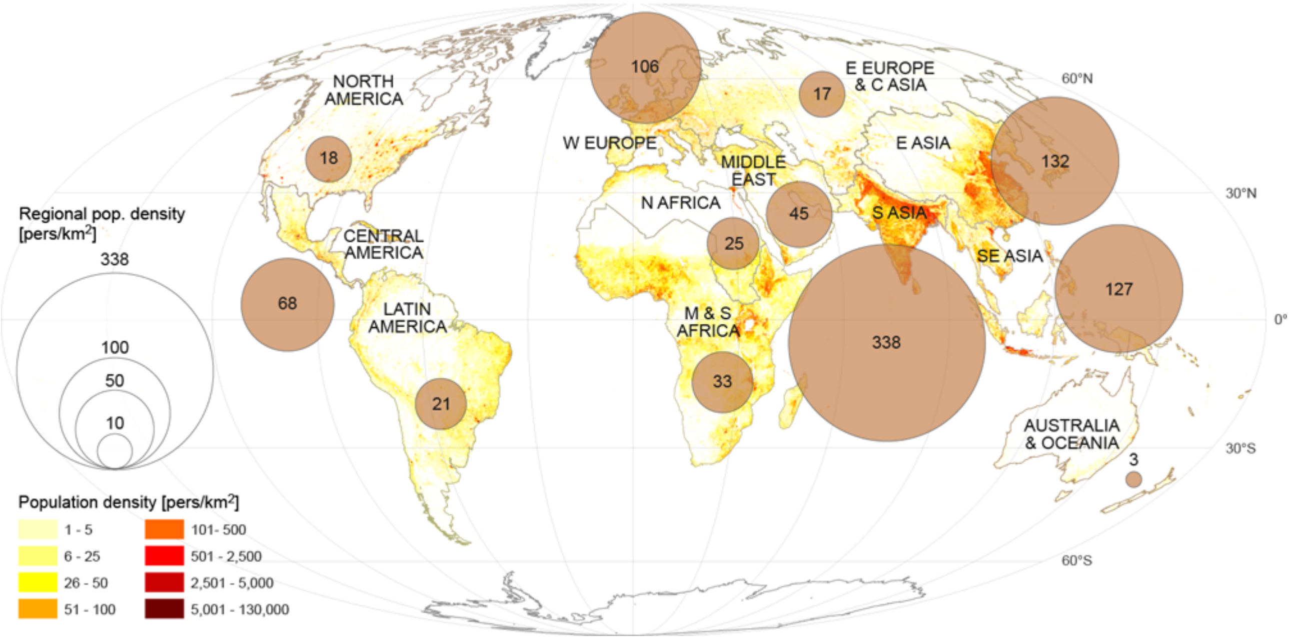

Figure S1. Population distribution across the globe (denoted by colour) (*note: grid cell size is 30″ by 30″, or approximately 1 km by 1 km at the equator*) and average regional population densities (denoted by circles).

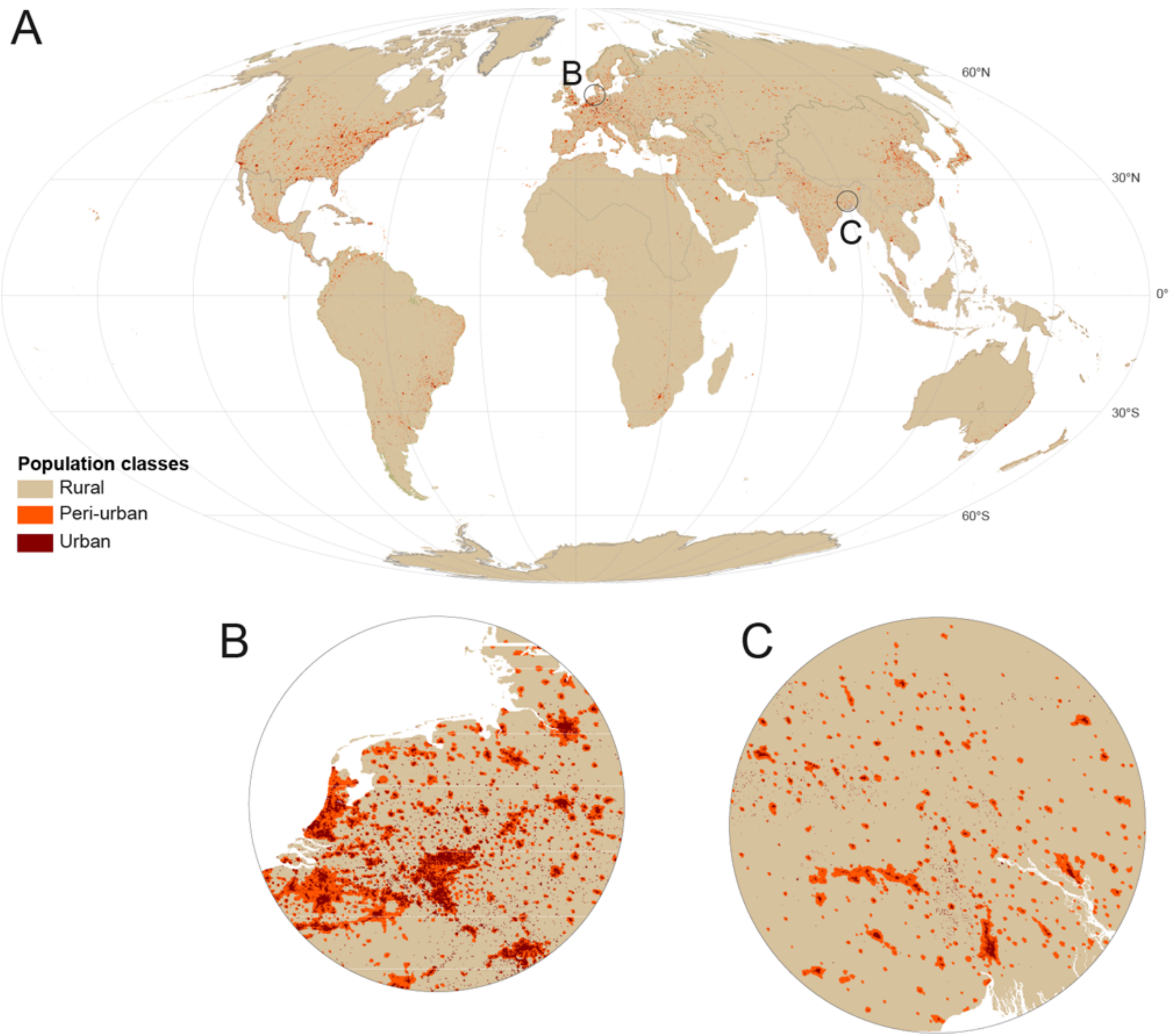

Figure S2. Map of the population classes (rural, peri-urban and urban): A. Global; B. part of northwest Europe; and C. Bangladesh and part of northeast India. See Table 1 and Section 2.1.2 in the article for the data sources.

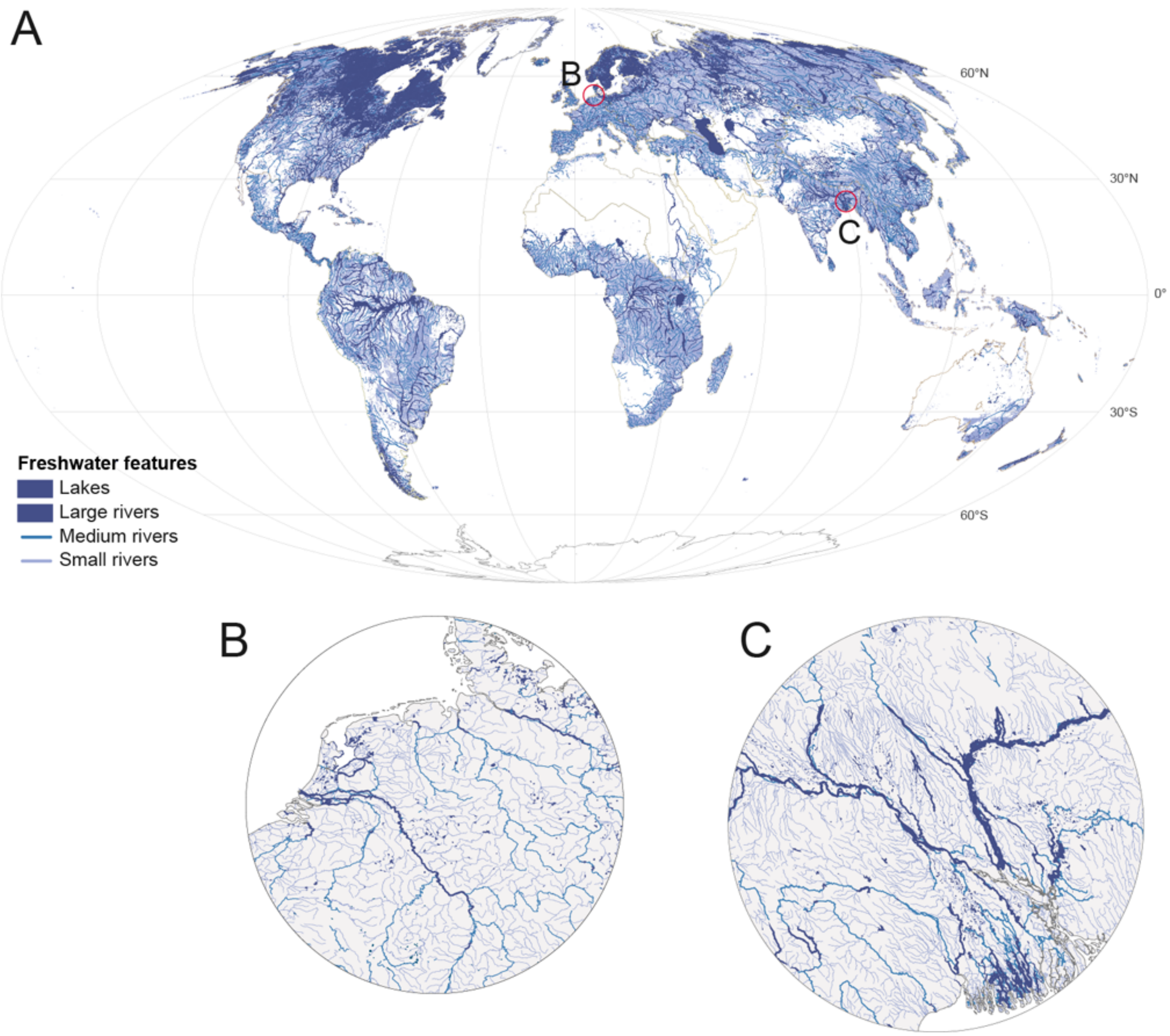

Figure S3. Map of the freshwater features used, namely: lakes, large rivers, medium rivers and small rivers: A. Global; B. part of northwest Europe; and C: Bangladesh and part of northeast India. Note: Only perennial rivers were included in the analysis and thus shown here. See Table 1 and Section 2.1.2 in the article for the data sources.

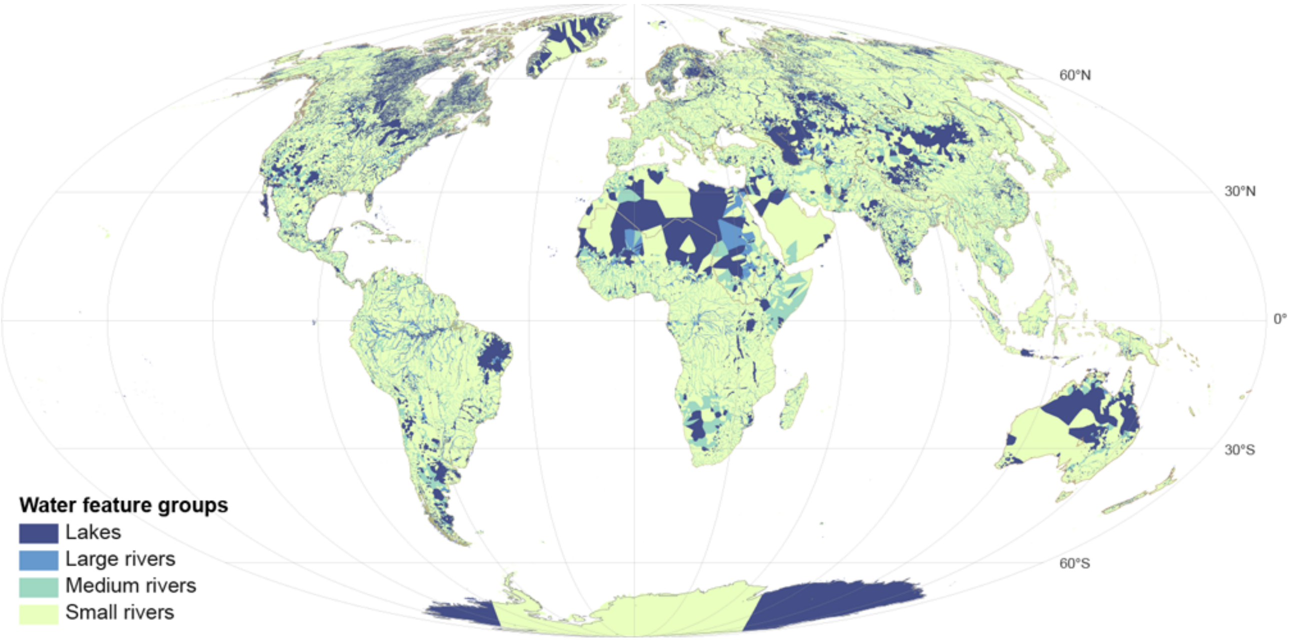

Figure S4. "Water feature map" showing, for each grid-cell, the class of the freshwater body (lake, large river, medium river, and small river) closest to it.
