# Supplementary material for: How Close Do We Live to Water? A Global Analysis of Population Distance to Freshwater Bodies
Source: PLoS One. 2011 Jun 8;6(6):e20578. doi: 10.1371/journal.pone.0020578 (PMC3110782; doi:10.1371/journal.pone.0020578)
Supplement: Supporting Information S2 — Supplement for Results section. (PDF) [file pone.0020578.s002.pdf]

Supporting information S2: Supplement for Results section

of the article: *Kummu et al. 2011. How close do we live to water? A global analysis of population distance to freshwater bodies,* published at PLoS ONE

S2.1 Water feature groups

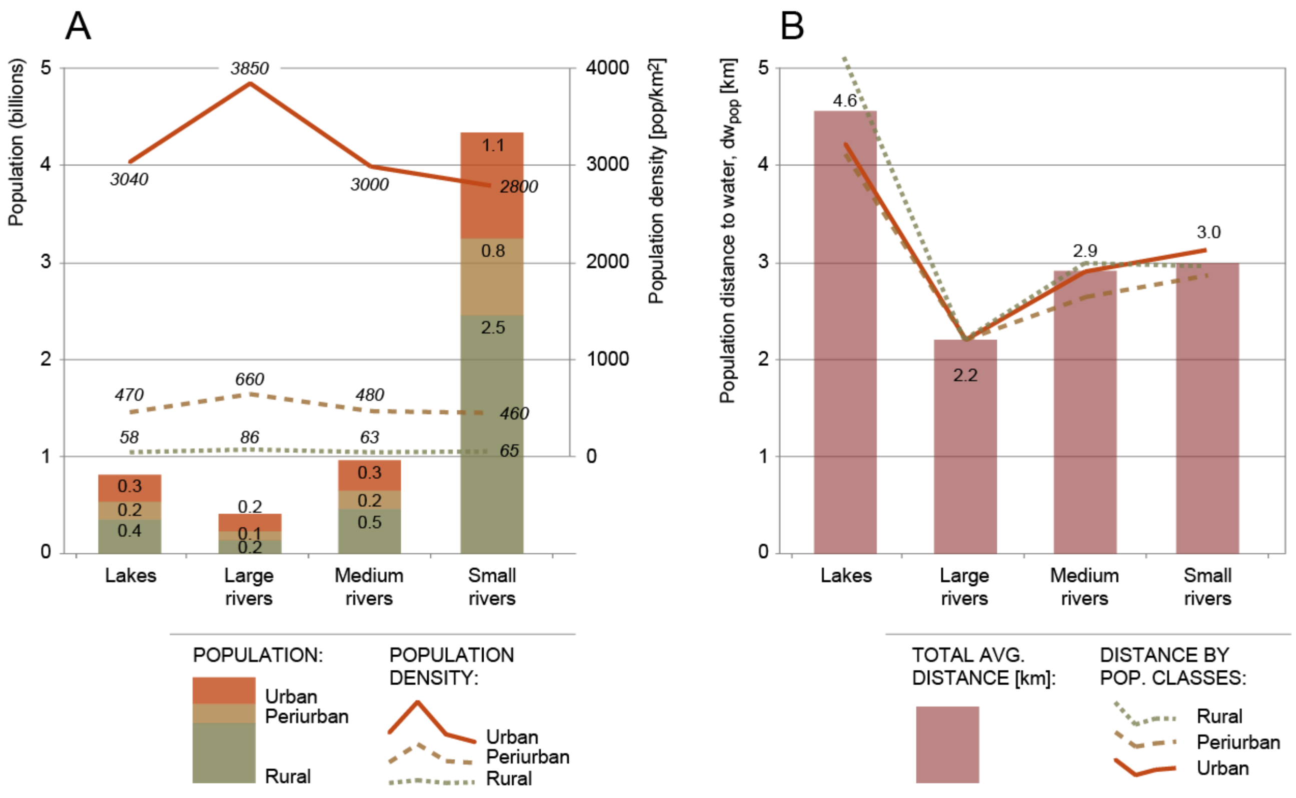

Figure S5. Results for water feature groups. A: Population and population density divided by the water feature groups (i.e. the closest water body of that certain land unit); and B: Population distance to water (dw_{pop}) divided by water feature groups.

S2.2 Country results

Of the analysed countries, people in Suriname live on average the closest to water (1.6 km), followed by Tajikistan and Kyrgyzstan where the average distance to water is 1.9 km from water (Figure S6). People in Libya (233 km) and Saudi Arabia (223 km) live the furthest from water (Figure S6). In half of the twelve regions, the median distance to water was 5

km or less in all countries. The larges variation in distance to water is in Middle East and North Africa, where the distance ranges from ca. 2 km up to 220-230 km (Figure S6).

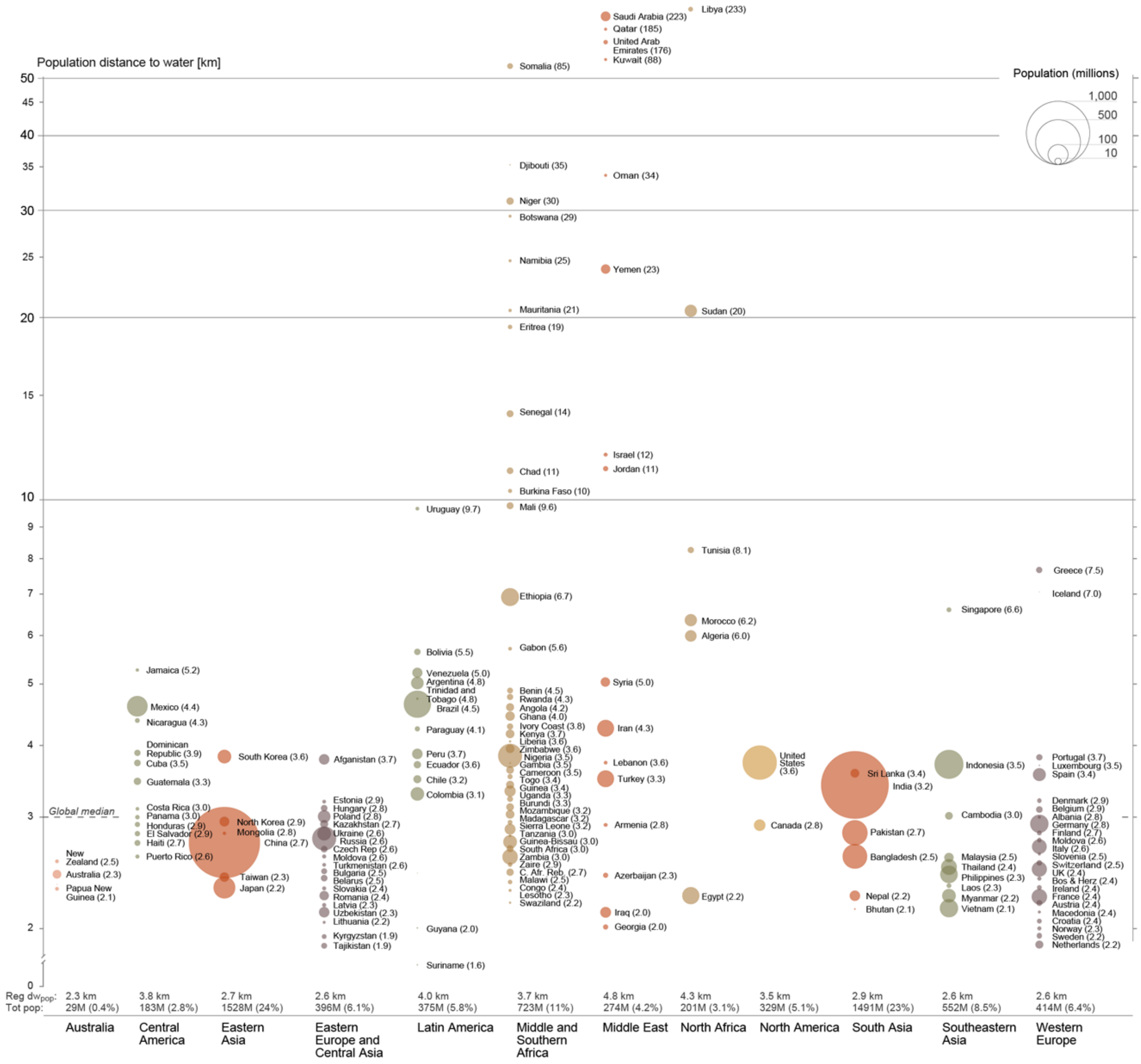

Figure S6. Country scale results for median dw_{pop} (population distance to water), categorised by regions. The sizes of the circles are proportional to population, based on LandScan 2007 data. The dw_{pop} [km] is indicated in brackets after the country name; global median dw_{pop} is 3.0 km. Regional average distances and total populations are presented at the bottom of the graph. Note: the y-axis is on a logarithmic scale.

S2.3 Statistical relationships

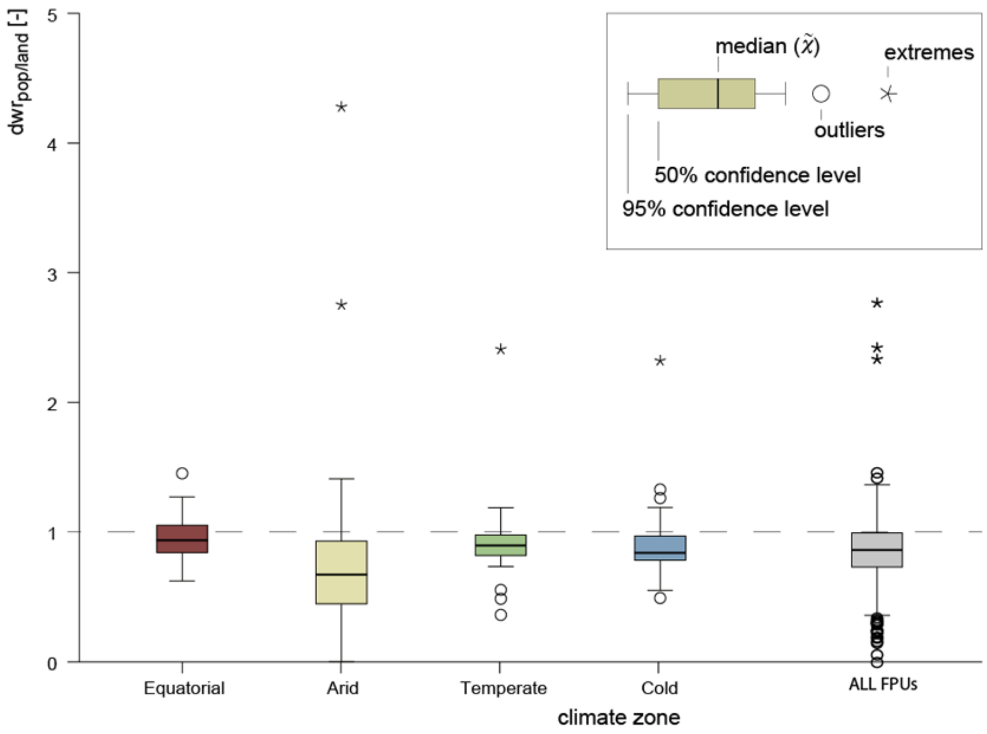

Figure S7. Box-plot of the FPU scale $dwr_{pop/land}$ (ratio of population distance to water over land distance to water) for each climate zone, and for all the FPUs together.

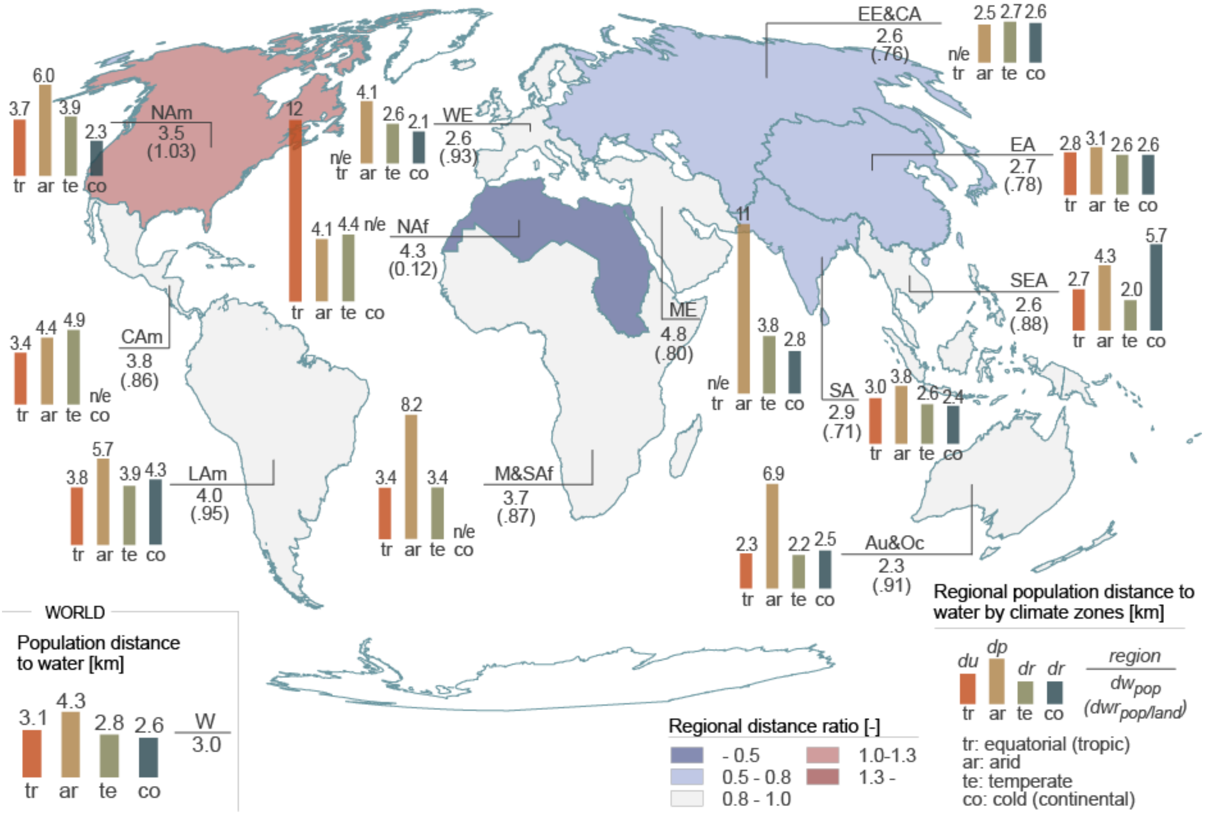

Figure S8. Regional results for the population distance to water (dw_{pop}) per climate zones (equatorial, arid, temperate, and cold), presented as columns. The regional ratio of population distance to freshwater over the land distance to water ($dwr_{pop/land}$) is presented as colours in the map (see also Figure 5 in the main article). Note: the abbreviations for the regions are as follows: Au&Oc-Australia and Oceania; CAm-Central America; EA-Eastern Asia; EE&CA-Eastern Europe and Central Asia; SA-South Asia; LAm-Latin America; ME-Middle East; M&SAf-Middle and Southern Africa; NAf-North Africa; NAm-North America; SEA-Southeast Asia; and WE-Western Europe.

We found significant bivariate correlation between $dwr_{pop/land}$ and aridity (Table S1 and Figure S9). This indicates that the more arid the region is, the more concentrated the population is close to water bodies (i.e. people live relatively closer to water compared to the available water features in that area).

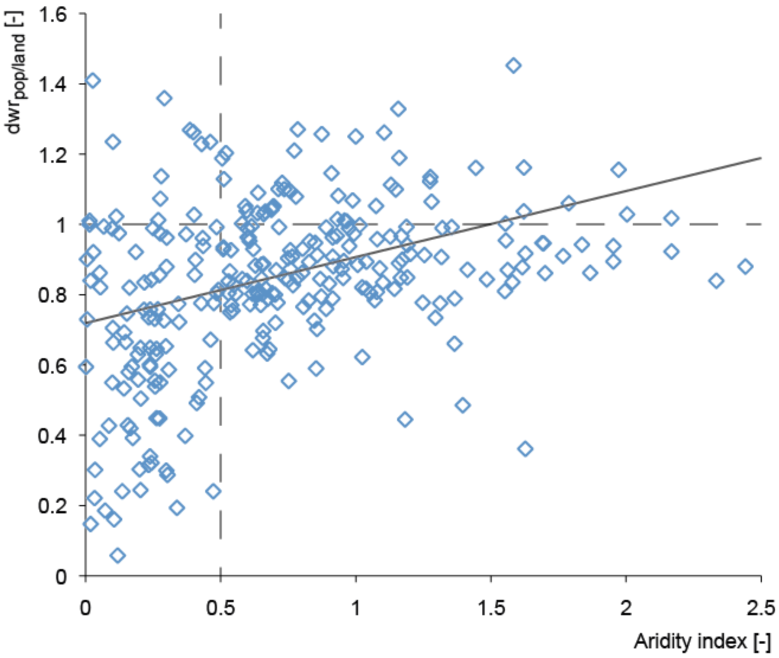

Figure S9. Scatter plot of aridity index vs. $dwr_{pop/land}$ (ratio of population distance to water over land distance to water) with linear trend line. According to the aridity index classification (Trabucco and Zomer, 2009), index values below 0.2 represent arid climates while values between 0.2 and 0.5 represent semi-arid climates.

Table S1. Bivariate correlations between the selected variables.

Correlations

		Median Distance to Water per person	Median Distance to water per cell	Water Availability per person	Precipitation	Avg Temp	Population density	aridity	dist_ratio
Median Distance to Water per person	Pearson Correlation	1	.923**	-.024	-.258**	.186**	-.065	-.221**	.200**
	Sig. (2-tailed)		.000	.685	.000	.002	.273	.000	.001
	N	284	284	284	284	284	284	284	284
Median Distance to water per cell	Pearson Correlation	.923**	1	-.009	-.311**	.217**	-.085	-.285**	-.002
	Sig. (2-tailed)	.000		.878	.000	.000	.152	.000	.971
	N	284	285	285	285	285	285	285	285
Water Availability per person	Pearson Correlation	-.024	-.009	1	-.056	-.081	-.041	-.043	.038
	Sig. (2-tailed)	.685	.878		.343	.174	.492	.469	.523
	N	284	285	285	285	285	285	285	285
Precipitation	Pearson Correlation	-.258**	-.311**	-.056	1	.384**	.180**	.745**	.203**
	Sig. (2-tailed)	.000	.000	.343		.000	.002	.000	.001
	N	284	285	285	285	285	285	285	285
Avg Temp	Pearson Correlation	.186**	.217**	-.081	.384**	1	.098	-.077	-.016
	Sig. (2-tailed)	.002	.000	.174	.000		.099	.194	.789
	N	284	285	285	285	285	285	285	285
Population density	Pearson Correlation	-.065	-.085	-.041	.180**	.098	1	.142*	.072
	Sig. (2-tailed)	.273	.152	.492	.002	.099		.017	.224
	N	284	285	285	285	285	285	285	285
aridity	Pearson Correlation	-.221**	-.285**	-.043	.745**	-.077	.142*	1	.272**
	Sig. (2-tailed)	.000	.000	.469	.000	.194	.017		.000
	N	284	285	285	285	285	285	285	285
dist_ratio	Pearson Correlation	.200**	-.002	.038	.203**	-.016	.072	.272**	1
	Sig. (2-tailed)	.001	.971	.523	.001	.789	.224	.000	
	N	284	285	285	285	285	285	285	285

**. Correlation is significant at the 0.01 level (2-tailed).
*. Correlation is significant at the 0.05 level (2-tailed).

We also divided the arid zone into five geographical regions (see Table S2) and performed the same regression analyses as presented above, in order to find possible regional differences within the arid zone. According to our analysis, the physical factors can neither explain the population density in Australia and America, nor in the Middle East (Table S2). In Northern Africa, combinations of climate variables and distance to water show significant correlation with population density; neither climatic parameters nor distance to water alone show significant correlation (Table S2).

Middle and Southern Africa is the only region in which population density in arid FPUs is significantly correlated with dw_{land} alone; a significant bivariate relationship was also found between population density and precipitation (Table S2). All of the combinations of independent variables also correlate significantly with population density, except aridity & temperature. In Asia, the population densities are explained very well by either precipitation or temperature (Table S2), whilst distance to water alone does not provide significant correlation.

Table S2. Results of the multiple regression analysis in the arid zone. The dependent variable is population density; the predictor(s) in each case are listed in the first column. The analysis was carried out for FPUs where the dominant climate zone is arid. Results are presented for the whole globe and then separately for each region (Aus&Am includes all the regions in America and Australia & Oceania; Asia includes Eastern Asia, South Asia and Eastern Europe and Central Asia). Note: In Western Europe and Southeastern Asia there are no FPUs where the spatially dominant climate zone is arid.

Variable	Globe (n=95)	Aus&Am (n=12)	Asia (n=21)	M&S Afr (n=39)	Middle East (n=9)	North Afr. (n=14)
dw_{land}	.096	.342	.164	.010**	.986	.104
Aridity	.205	.880	.122	.363	.539	.182
Prec	.112	.856	.000***	.000***	.696	.181
Temp	.901	.177	.001***	.488	.921	.965
dw_{land}& aridity	.086	.526	.155	.017*	.768	.025*
dw_{land}& prec	.008**	.627	.000***	.001**	.897	.004**
dw_{land}& temp	.198	.317	.001*	.015*	.991	.227
Aridity & prec	.216	.984	.000***	.001**	.396	.383
Aridity & temp	.448	.423	.002**	.510	.804	.427
Prec & temp	.239	.303	.000***	.001**	.928	.420
dw_{land}& aridity & prec	.018*	.736	.000***	.004**	.609	.016*
dw_{land}& aridity & temp	.146	.580	.001**	.017*	.920	.037*
dw_{land}& prec & temp	.002**	.517	.000***	.002**	.978	.006**
Aridity & prec & temp	.348	.370	.000***	.003**	.221	.607

*: p<0.05; **: p<0.01; ***: p<0.001

S2.4 Water shortage in relation to dw_{pop}

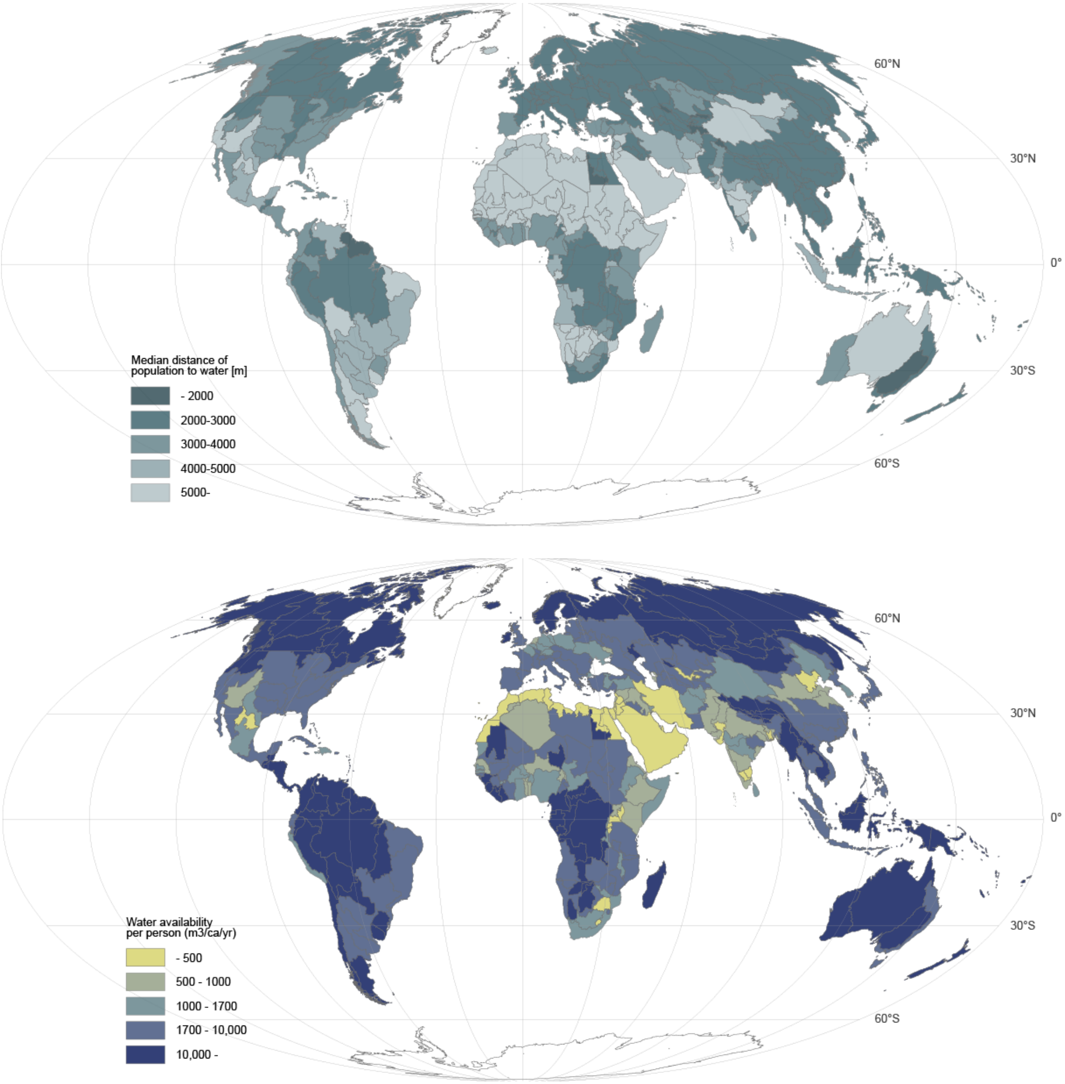

Figure S10. FPU scale results for population distance to water (dw_{pop}) (above); and water availability per person (below). Source for the water availability data: Kummu et al. (2010).
